# Evolution, ovulation and cancer

**DOI:** 10.7554/eLife.00729

**Published:** 2013-04-16

**Authors:** K VijayRaghavan, Satyajit Rath

**Affiliations:** Tata Institute of Fundamental Research, National Centre for Biological Sciences, Bangalore, Indiavijay@ncbs.res.in; National Institute of Immunology, New Delhi, Indiasatyajit@nii.res.in

**Keywords:** ovulation, sperm storage, exocrine gland, nuclear receptor, Notch signaling, cancer, *D. melanogaster*

## Abstract

Secretions by epithelial cells of the fallopian tube regulate ovulation through conserved pathways, which means that experiments on flies might provide insights into the human reproductive system and, possibly, ovarian cancer.

**Related research article** Sun J, Spradling AC. 2013. Ovulation in *Drosophila* is controlled by secretory cells of the female reproductive tract. *eLife* **2**:e00415. doi: 10.7554/eLife.00415

**Image** Glandular secretions from the *Drosophila* female reproductive tract promote ovulation and the storage of sperm in the spermatheca (pictured)

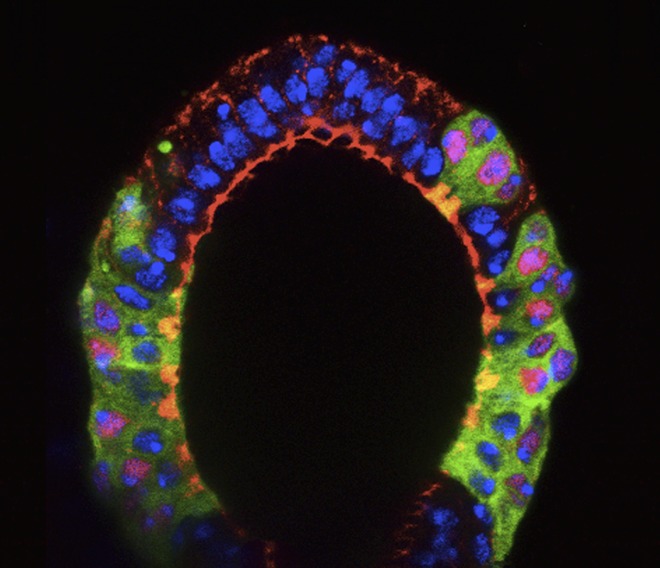

Cancer of the ovaries is difficult to detect early, hard to treat, and its origins are still controversial. Now, in *eLife*, Jianjun Sun and Allan Spradling of the Carnegie Institution for Science report new data on an evolutionarily conserved pathway for the regulation of ovarian function in the fruit fly (*Drosophila melanogaster*), which could advance our understanding of ovarian cancer in humans (Sun and Spradling, 2013).

As with breast cancer, a woman's risk of developing ovarian cancer is increased if she inherits mutant forms of the genes *BRCA1* and/or *BRCA2*. Some women with these mutations choose to have surgery to reduce their cancer risk (Berns and Bowtell, 2012), and studying tissues from these patients has increased our understanding of how the disease progresses. These and other studies have classified ovarian cancers into distinct types, and have also provided cell lines that can be used to study the disease at the molecular and cellular level (Levanon et al., 2010).

Some such studies suggest that cancers with an apparent ovarian origin—particularly high grade serous ovarian cancer—may in fact be cancers of the secretory epithelial cells of the fallopian tube. However, there is evidence that these cells may have an indirect role in some ovarian cancers too; simply tying off, or ligating, the fallopian tubes, which convey eggs from the ovaries to the uterus, decreases the incidence of ovarian cancer (Cibula et al., 2011). Moreover, there is other evidence for a link between ovarian cancer and the regulation of ovulation (Purdie et al., 2003). Although the major hormonal cues for ovulation are well characterized, the role of secretions from epithelial cells lining the fallopian tube in ovulation is poorly understood. However, if these pathways and molecules are conserved between species, the work of Sun and Spradling in *Drosophila* could allow us to make significant headway in understanding the secretory regulation of ovulation in mammals, and possibly shed new light on the genesis of ovarian cancers.

In both flies and mammals, ovulation usually results in the release of just one egg cell per cycle. In *Drosophila*, muscle contractions move the egg through the oviduct, where it can be fertilized by sperm stored in the spermathecae, before moving on to the uterus and finally being deposited from the vulva (Figure 1; Middleton et al., 2006). The work of Sun and Spradling builds on their previous research into the development of secretory glands in other parts of the female reproductive system (Allen and Spradling, 2008; Sun and Spradling, 2012). These studies identified the regulatory protein lozenge, which is a member of an evolutionarily conserved family of proteins called Runx, as an important player in secretory system development. And indeed, other members of the Runx family have been implicated in many ovarian cancers (Nevadunsky et al., 2009; Li et al., 2012; Keita et al., 2013).

**Figure 1.**
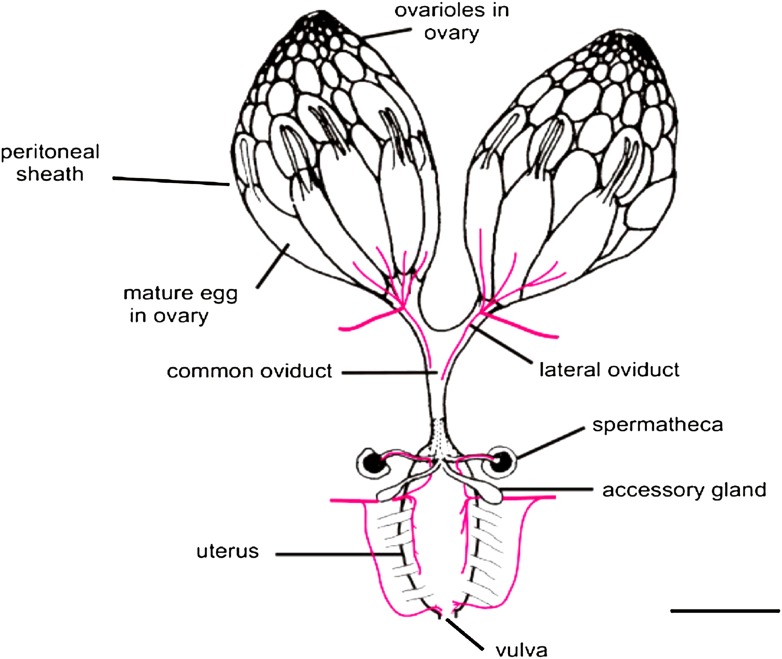
The *Drosophila* female reproductive tract (figure adapted from Middleton et al., 2006). The mature egg is transported through the oviduct during ovulation and is fertilized by sperm stored in the spermathecae and seminal receptacle (the latter is not shown). The spermathecae and the accessory glands (parovaria) are the major secretory components of the female reproductive system. Sun and Spradling (2013) show that glandular secretions from these structures regulate ovulation and promote sperm storage. The innervation of muscles is shown in magenta. Scale bar: ∼250 μm.

IMAGE: Middleton et al. (2006).

Sun and Spradling's experiments now tease apart the multiple roles of the secretory glands to home in on those aspects relevant to sperm storage and ovulation. They do this in two ways. First, they alter the number of secretory cells in the reproductive tract and show that there are adverse consequences for sperm storage and sperm health, as well as for ovulation. In fruit flies, sperm are stored inside the spermathecae and the seminal receptacle within a few hours of mating. Sun and Spradling find that reducing the number of secretory cells (to fewer than 25) prevents the accumulation of sperm in the spermathecae but not in the seminal receptacle, suggesting that distinct mechanisms target and/or retain sperm in each location. However, even sperm that do accumulate in the receptacle aggregate abnormally, showing that secretions are also essential for the health of the stored sperm.

When Sun and Spradling modulate well-known ‘canonical' molecular pathways controlling secretion in these cells, sperm storage in the spermathecae is reduced. However, abolishing these canonical secretory pathways does not affect ovulation, even though a reduction in the number of secretory cells does; this suggests that the effect of altering secretory cell number on ovulation is likely to be mediated by one or more secretions through non-canonical pathways.

These findings raise important questions about our understanding of ovarian function and cancer. Does the number and/or secretory ability of fallopian tube epithelial cells correlate with either ovulatory capacity or the ability of tubal epithelium to bind and retain sperm, and could this explain some types of infertility? Might the secretory ability of cells within the female reproductive system be linked to their reported high propensity to DNA damage, and so directly mediate their contribution to ovarian cancer? Also, if the number of secretory cells and/or the integrity of non-canonical secretory pathways are regulators of ovulation, abnormalities in these may contribute to abnormal ovulation, which could, in turn, influence the likelihood of cancer.

Consistent with an indirect role for secretory cells in some ovarian cancers, it is noteworthy that members of the Runx family of gene regulatory proteins, familiar figures in ovarian cancers, are reported to regulate secretion (Little et al., 2012). Such a possibility would connect elegantly with the reduced frequency of ovarian cancers upon fallopian tube ligation. But which genes and proteins control these secretory pathways? There are now enough genetic and molecular tools available—in both fruit fly and mammalian systems—to make identifying these components a tractable endeavor. Isolating secretions from mammalian fallopian tubes and examining their effects on *Drosophila* ovulation could be a fruitful exercise, as could genetic approaches that screen directly for ovulation defects. The work of Sun and Spradling thus opens up new avenues for the genetic and molecular analysis of the properties and functions of secretory cells in the reproductive tract, and their potential role in evolutionarily conserved aspects of carcinogenesis.
